# A critical appraisal of the ENLIST severity scale for erythema nodosum leprosum

**DOI:** 10.1371/journal.pntd.0010378

**Published:** 2022-04-26

**Authors:** Bhushan Kumar, Hitaishi Mehta, Tarun Narang, Sunil Dogra

**Affiliations:** 1 Senior Consultant, Department of Dermatology, Shalby Hospital, SAS Nagar, Punjab, India; 2 Department of Dermatology, Venereology and Leprology; Postgraduate Institute of Medical Education and Research; Chandigarh, India; Hospital Infantil de Mexico Federico Gomez, MEXICO

During the process of revision of IAL Textbook of Leprosy (3^rd^ Edition) we had the occasion to look more carefully at the 10-item ENLIST-ENL severity scale for erythema nodosum leprosum (ENL) which is now being followed by many researchers in many countries (Available from: https://bit.ly/3uc1pYt) [1,2]. The scale was essentially developed to fulfill a need- because as per the authors the previously available scales were not properly validated [1,3–5].

To reiterate the well-known definition of ENL as given by the study group: ENL causes inflammation in many systems and is characterized by severe pain, tender cutaneous lesions, fever, joint pains, bone pain, eye inflammation, orchitis, lymphadenopathy, neuritis, peripheral oedema and renal involvement etc. Involvement of liver and adrenals has also been reported. It is obvious that some symptoms are statistically more common (all types of pains, fever, lymphadenopathy, peripheral edema) but may be less damaging and some are less common but of greater significance and consequences (neuritis, iritis, orchitis, renal involvement, ulcerative / necrotic lesions of ENL). However, the authors followed the approach which was more statistically relevant rather than based on the severity/sequelae of the complications. Based on the concept of minimal important difference (MID) of clinical relevance to the outcome, the authors excluded the items showing lowest level of correlation viz. inflammation of the eyes, urine analysis, orchitis and items related to sensory and motor nerve function impairment (NFI) from the final version, as these were documented to result in reduction in internal consistency calculated using Cronbach’s alpha [2]. Cronbach’s alpha determines how closely the items in a scale are related to each other. Out of the 10 items, 3 pertain to skin lesions (item 3, 4, 5) and three more are related to pain (item 1, 7, 10). Redundancy of parameters may have resulted in a high alpha value, leading to overestimation of the reliability of this scale. As multiple factors (i.e., various organ system involvement) underlie the severity of ENL, reliance on Cronbach’s alpha as the sole measure of internal consistency does not seem justified [6–8].

Neuritis / nerve pain is very frequent complaint of patients with ENL. To emphasize on the significance of the nerve involvement; in the INFIR study (quoted also by the authors) individuals with ENL had evidence of significant subclinical nerve involvement on sensory and motor nerve conduction and warm detection threshold [9]. In a study on ENL from Nepal, 99% of the patients had enlarged or tender nerves and new and/or old NFI was reported in 88% of the patients [3]. In the concluding paragraph of their own publication in 2015, the authors also agreed that ‘ENL is associated with a high prevalence of neurological impairment and poses a considerable management problem’ [1].

Even when 22.9% of their patients met the criteria of new NFI and 7.9% of the patients presented only with neuritis, the authors still decided to ignore this important component of type 2 reaction for grading of severity. The authors considered this as of no consequence / clinical relevance, even when many of their Ethiopian patients (part of the study) had ENL related nerve tenderness and met the criteria of new NFI. So, focusing on a single neural symptom while disregarding the other does not take away the seriousness of the problem of an individual patient. Instead, the current score assigns a unreasonably high weightage to skin lesions (3 out of 10 items) as compared to the nerve involvement, although the latter has longer and more severe implications of the overall health status as well as the outcome.

The authors have included ‘neuritis’ as an item only for scoring for pain without considering that severe inflammation produces acute neuritis and the consequential severe pain, indicating severe nerve involvement which is bound to end up in most cases in nerve deficit, sensory, motor or both. A person who has severe grade of ENL is sick–with severe and distressing nerve pain- so evaluation for NFI may not be easy or exact at the time of acute illness- only on the subsidence of nerve pain the NFI can be more clearly appreciated.

Taking an example, if a patient has only severe neuritis, no skin lesions of ENL, the maximum score will only be 4, whereas the patient technically falls into the category of severe type 2 reaction (ENL without skin lesions). Even if we add another 3 or 4 points for pain present anywhere else and of a different kind the score will never reach 9—the critical cut off point to make it eligible for the label of ‘severe’ reaction. The authors were aware of such a situation and so did add it in the inclusion criteria, “In the absence of cutaneous signs ENL could also be diagnosed if a patient with leprosy had fever or malaise and histological features consistent with ENL in a tissue biopsy” [1]. Why they decided to stop at that is not understood.

Although the seriousness of an inflammatory response is related to the severity of damage to the important / vital organ(s) it can/ will produce, their percentage and statistical significance are of no concern to the patient who suffers from it. A person with severe neuritis, orchitis, iritis, ulcerative/necrotic lesions of ENL is more likely to end up with a serious damage to an important organ of consequence. Loss of eyesight, development or worsening of the deformity, increased morbidity or even mortality due to septicemia developing due to ulcerated and necrotic lesions is indeed serious. The items obviously have been chosen not on their significance but their prevalence–lymphadenitis in 14.7% was chosen over orchitis occurring in 13.5%. Same is true about the inclusion of peripheral edema. Though temporarily distressing /even incapacitating edema is the first to settle leaving behind hardly any residue.

So the issues we wish to raise pertain to the significance/importance of an ‘item’ / symptom/sign for inclusion, pattern of scoring/grading and definitions of the terminologies used by authors.

1. In the morphology of the lesions- the authors have quoted the figure for vesicles, bullae or pustular lesions to be 14%- but in tabular representation of the data pertaining to clinical features of ENL, the figures for pustules, vesicles, bullae, necrotic and ulcerated lesions add up to (110/292) more than 30% which is not an insignificant number, whatever the statistical calculations.
2. Pain: The ‘pain’ at item No.1 probably means ‘pain in the skin or pain in the nodules of ENL’. Bone pain (item 7), arthritis, pain of neuritis (item 10) and the other unspecified pains- backache, neck pain, muscle pain and headache etc., and how to exactly differentiate one from the other is not mentioned. The compartmentalization of pain is not so clear cut in a sick patient–there is always an overlap. Moreover, the authors themselves while referring to a study on ENL from Nepal [3] where joint pain, nerve pain and muscle pain were itemized separately, had commented “it is not clear how these were defined nor whether these symptoms were solely attributable to ENL”? “The differences between the two studies are likely to be due to the methodological differences employed” [1]. There is not likely to be any differences (methodological) between the ‘sites’ of pain as complained by the patient- only difference could be in the grading of pain which the study from Nepal did not undertake.
3. Item No.2: Fever. It has been considered as an item of significance even when in actuality only 19.8% had fever as documented and “there was no significant difference in the grade of temperature between individuals with different types (grades) of ENL”. The patient had ‘feeling of fever’- moreover, all these feelings of weakness, lassitude, difficulty in even getting up, sick look and other feelings of “not well” are due to the release of inflammatory cytokines (some of which are pyrogenic)-but they do not always produce corresponding degree of fever. Some precipitating factors like viral or bacterial infections could also be responsible for the added spike. Depression present in majority of the patients also adds to the feeling of being unwell- fever may not be the only cause.
4. Item No. 3, 4 and 5, referring to the number of lesions, inflammation of the lesions and even the related pain in the ENL nodule and extent of skin involvement and occurrence of vesicles, pustules, panniculitis etc. are interrelated and constitute a spectrum. More severe inflammatory process will have more number of skin lesions, larger area of involvement and more severe symptoms. Grading skin lesions by three different measures may not give us any additional information.
5. Item No.9: Lymphadenopathy: what would the term ‘enlarged’ (increased in the earlier version) mean, as lymphadenopathy by definition means enlarged lymph node(s). So, the heading under the score of 1 in this criteria is confusing.
6. Headings of some of the items listed for scoring is ambiguous i) Item No.4: inflammation of lesions- ‘absent’, ‘erythematous’, ‘painful’ and ‘complex’. All of them do not convey the same meaning in a continuum. ‘Absent’ inflammation in a lesion of ENL (which by definition is an inflammatory lesion) is implausible. ‘Complex’ in an earlier publication has been described to mean vesicular, bullous, pustular, erythema multiforme-like, panniculitis, necrotic, ulcerated". However, erythema multiforme-like lesions are a morphological variant and does not indicate severity.
7. Even when it is a cross-sectional one-time study- the authors after accepting the observations of the various other studies that “ENL manifests broadly the same way at different centers” express reservations about the observations in other studies “but the inferences are to be treated with caution due to confounding factors” which essentially are the same as reported by the authors themselves.
8. More importantly, the grading of reactions as mild, moderate or severe was done by different observers across the study centers and the individual subjectivity may have hampered the objective grading.

We would suggest that in the next revision of this useful scale, the authors should consider adding that presence of iritis/ iridocyclitis, orchitis, ulcerated/ necrotic skin lesions, and severe neuritis (even in the absence of skin lesions), whatever may be the score, should be labeled as severe ENL. This approach is similar to the lines on which authors have decided to categorize chronic ENL under ‘severe’ disease (even when the symptoms and signs do not qualify for that label) being fully aware that the episode of reaction is not always ‘severe’ in degree–; it is the sequelae that are of importance even if they affect a minority of the patients. So, for scoring, it should not matter when an important/ vital organ is involved in 1in 10 or 1 in 1000 patients. Further, various ambiguities in the terminology for scoring should be clarified–if the scale is intended to be ultimately used by health workers, physiotherapists or general duty medical officers involved in care of leprosy affected people.
